# Single-port robot-assisted perineal radical prostatectomy with the da Vinci XI system: initial experience and learning curve using the cumulative sum method

**DOI:** 10.1186/s12957-023-02927-9

**Published:** 2023-02-13

**Authors:** Chenhao Yu, Li Xu, Liyin Ye, Qiming Zheng, Haiyi Hu, Kangxin Ni, Chenghao Zhou, Dingwei Xue, Sheng Cheng, Hui Wang, Raymond Wei Pak, Gonghui Li

**Affiliations:** 1 Department of Urology, Sir Run Run Shaw Hospital, Zhejiang University School of Medicine, No. 3 East Qingchun Road, Hangzhou, 310016 Zhejiang China; 2 Department of Urology, Mayo Clinic-Jacksonville, 4500 San Pablo Road, Jacksonville, FL 32224 USA

**Keywords:** Prostate cancer, Radical prostatectomy, Perineal prostatectomy, Single port, Learning curve

## Abstract

**Background:**

To evaluate the early functional and oncological outcomes of single-port robot-assisted perineal radical prostatectomy (sp-pRARP) using the da Vinci XI system and analyze its learning curve using the cumulative sum (CUSUM) method.

**Methods:**

The clinical data of 50 patients who underwent sp-pRARP for localized prostate cancer between May 2020 and May 2022 in our center by a single surgeon were analyzed retrospectively. Demographic information, preoperative and postoperative variables, complications, early functional and oncological outcomes of patients were recorded. The CUSUM method was used to illustrate the learning curve based on operation time.

**Results:**

All surgeries were completed without conversion. The median (interquartile range, IQR) operation time was 205.0 (82.5) min, whereas the median (IQR) docking time was 30.0 (15.0) min and the console time was 120.0 (80.5) min. The median (IQR) estimated blood loss (EBL) was 50.0 (137.5) mL. Positive surgical margins were detected in five patients (10.0%). The continence rate was 40.9%, 63.6%, 88.4%, and 97.7% at the 1, 3, 6, and 12 months after surgery. According to the CUSUM plot, the inflection points of the learning curve were 20 cases, splitting the case series into “early phase” and “late phase.” In “late phase” cases, there was less time spent on each step of the operation and less EBL.

**Conclusions:**

Sp-pRARP using the da Vinci XI system was verified to be a feasible and reliable surgical approach. According to the CUSUM plot, 20 cases was considered the turning point for surgeons to master the novel technique.

**Supplementary Information:**

The online version contains supplementary material available at 10.1186/s12957-023-02927-9.

## Background

Radical prostatectomy (RP) is the standard of care for the surgical treatment of localized prostate cancer (PCa). Young [1] first described RP using the perineal approach in 1904, which remained the preferred technique for the next half-century. However, the anatomical complexity of the perineum and the relatively high frequency of serious complications, such as rectal injuries and fecal incontinence, rendered the procedure difficult and concerning to surgeons. Millin [2] pioneered a retropubic approach to RP in 1945, which increasingly supplanted the perineal approach.

With the development of minimally invasive technologies, surgeons now have a sharper field of vision and more user-friendly surgical instruments. In the era of robotic surgery, the transperitoneal, extraperitoneal, or transvesical techniques, which have similar efficacy, have been utilized for a variety of purposes [3, 4], and the perineal approach is also back in the spotlight. At the same time, several intraoperative techniques based on anatomy are applied to improve the functional outcomes [5]. In 2016, Kaouk et al. [6] first conducted single-port robot-assisted perineal radical prostatectomy (sp-pRARP) on four patients using da Vinci SI and XI systems. Encouraging findings supported the possibility of a robotic perineal approach and the latest da Vinci SP system, purposed built for single-port surgeries with an “elbow” structure and allowing a single arm to have 360° of anatomical access [7], made it more attractive to additional surgeons. Subsequently, Tugcu et al. [8],Vitarelli et al. [9], Chang et al. [10], and Lenfant et al. [11] reported their case series. These investigations all demonstrated that sp-pRARP was a feasible and safe approach.

However, no large-scale investigation of sp-pRARP using XI systems had been conducted. Our previous successful outcomes with nine PCa patients using the da Vinci SI system proved the safety and efficacy of sp-pRARP [12]. This retrospective study aimed to describe a cohort of patients who underwent sp-pRARP on the da Vinci XI system and to analyze the learning curve using the cumulative sum (CUSUM) method.

## Methods

### Patient selection

Patients who underwent sp-pRARP for localized PCa between May 2020 and May 2022 in our center were analyzed retrospectively. All patients were diagnosed with localized PCa by ultrasound-guided prostate biopsy, suprapubic ultrasonography, and contrast-enhanced prostate MRI. PET/CT was used to evaluate for lymph node metastasis in patients who were at a high probability of developing it. Patients with a large prostate (volume>80cc), lymph node metastases determined by MRI or PET/CT or other severe cardiopulmonary diseases who could not tolerate an exaggerated lithotomy position were excluded. All the surgeries were performed by one experienced surgeon (GH Li) whom completed over 1000 cases of multiport robot-assisted radical prostatectomy (mp-RARP) and 100 cases of single-port robot-assisted extraperitoneal radical prostatectomy (sp-eRARP).

### Surgical technique

**Fig. 1:**
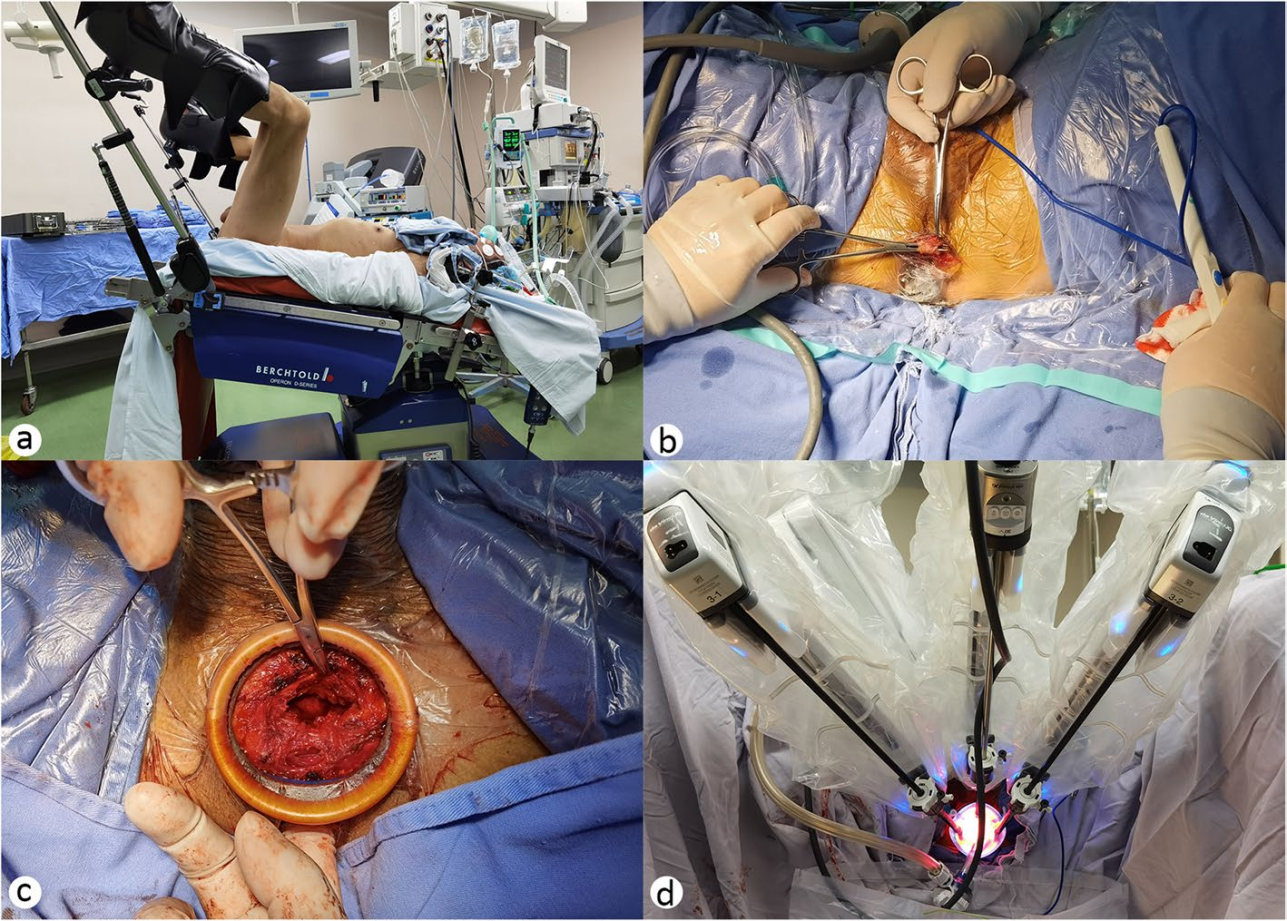
Patient positioning and robot docking. **a** Patient placed in a 20° Trendelenburg tilt. **b** Division of the perineal body. **c** Division of the recto-urethral muscles. **d** The placement of robotic arms

**Fig. 2:**
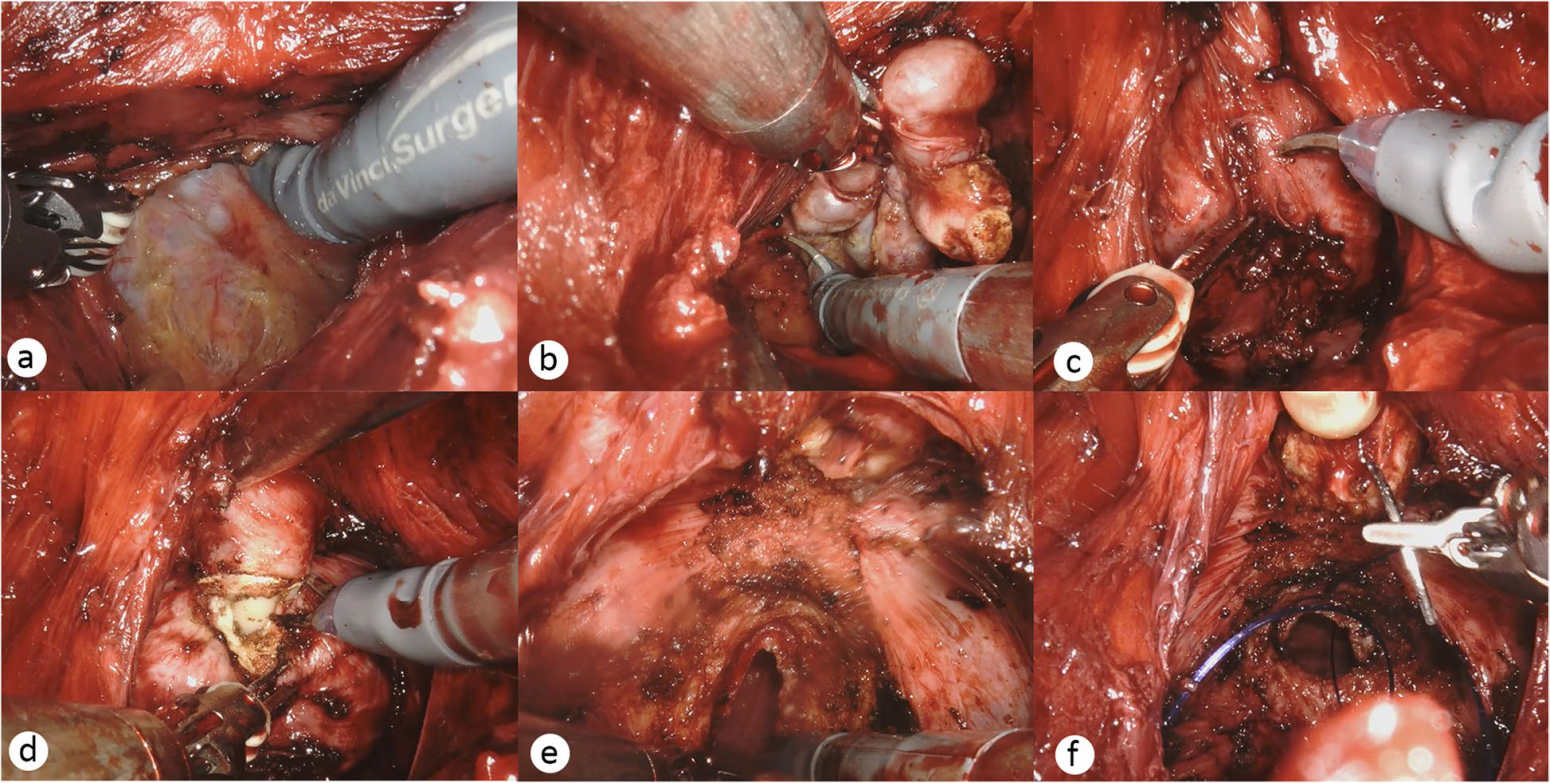
Surgical procedure. **a** Dissection of Denonvilliers’ fascia. **b** Exposure of bilateral seminal vesicles. **c** Blunt separation of the capsules on both sides of the prostate. **d** Dissection the apex of the prostate and cut the urethra. **e** Excision of the prostate and bladder neck dissection. **f** Vesicourethral anastomosis

An inverted U-shaped incision is made on the mid-perineum between the bilateral ischial tuberosities. After the subcutaneous fascia is separated, the central tendon is incised. Next, the rectourethral muscle is divided, and the space anterior to the rectum is developed by blunt dissection. The levator ani muscles are separated to expose Denonvilliers’ fascia. Then, the disposable multi-channel laparoscopic surgical access system is inserted with a surgical wound protector (Angel Medical Instruments Co., Ltd., Jiangsu, China) through the perineal incision. The intelligent pneumoperitoneum Airseal® insufflator (Surgiquest Inc., Milford, CT, USA) is connected for a stabilized pressure at 12 mm Hg to establish the pneumoperitoneum, and the trocars and camera port are placed in specific positions (shown in Fig. 1). Denonvilliers’ fascia is incised transversely below the prostate to expose the bilateral seminal vesicles and the ampulla of the vas deferens, which are subsequently divided. Blunt separation is performed on both sides along the capsule of the prostate, and then, the vascular pedicles of the prostate are ligated. The urethra is divided at the junction between the apex of the prostate and the membranous urethra. The apex of the prostate is retracted to separate the anterior prostate to the bladder neck, and then, the bladder neck is identified. The prostate and seminal vesicles are excised and removed en bloc. Finally, the vesicourethral anastomosis is performed using 3-0 bi-directional barbed sutures, and a Jackson-Pratt drainage tube is placed before incision closure (shown in Fig. 2).

### Data collection

Demographic information, preoperative and postoperative variables, complications, and functional and oncological outcomes of patients were recorded and analyzed. Preoperative estimated prostate volume was measured by transrectal ultrasound. Prostatectomy specimens were examined for final pathological staging and surgical margins. The pathological stage was carried out according to the 2017 TNM classification [13]. Complications were graded according to the Clavien–Dindo system [14]. The functional and oncological outcomes were evaluated at 1, 3, 6, and 12 months after surgery by evaluating the continence status and PSA blood test results. Return of urinary continence status was defined as using no more than one safety pad per day [15]. Biochemical recurrence was defined as a PSA level >0.2 ng/mL at two consecutive measurements [16].

### CUSUM analysis

CUSUM analysis was first described by Noyez [17] and describes the deviation between the data of individual cases and the average value of the overall data and then displays each deviation sequentially [18]. Operation time CUSUM was calculated as ${\sum}_{i=1}^n\left({x}_i-\mu \right)$, where *x*_*i*_ was operation time of each case and *μ* was the mean operation time of the cohort. The peak of the CUSUM curve was considered the threshold point for different phases of the surgery learning curve.

### Statistical analysis

Continuous variables conforming to a normal distribution were expressed as the mean ± standard deviation, and the independent-sample Student’s *t* test was used to analyze differences between the groups. Non-normally distributed data were presented with the median [interquartile range, (IQR)], and differences between groups were tested with the Mann-Whitney *U* test. Differences between categorical variables were assessed using the chi-square and Fisher exact tests. Statistical analysis was performed by Package for Social Sciences (IBM SPSS Statistics; New York, NY, USA, version 26.0). All tests were two-sided, with a significance set at *p* < 0.05.

### Ethics

The study was conducted in accordance with the Declaration of Helsinki (as revised in 2013) and was approved by the Ethics Committee of Sir Run Run Shaw Hospital, School of Medicine, Zhejiang University (2022- research-0207). The written informed consents were taken from all patients.

## Results

Fifty patients in total were included in this study. The mean patient age was 67.4±6.1 years and the mean body mass index (BMI) was 24.1±3.2kg/m^2^. The median (IQR) prostate volume was 32.6 (18.6) mL and the preoperative PSA level was 8.9 (6.3) ng/mL. Notably, 17 patients (34%) previously underwent a combined total of 22 abdominopelvic surgeries. The data of biopsy grade group, NCCN risk group, surgical history, American Society of Anesthesiologists (ASA), and Charlson comorbidity index (CCI) scores are listed in Table 1.

**Table 1:** Patient demographics (*N*=50)

Variables	Results
Age, years	67.4±6.1
BMI, kg/m^2^	24.1±3.2
Prostate volume, mL	32.6 (18.6)
Preoperative PSA level, ng/mL	8.9 (6.3)
Clinical stage, *n* (%)	
T1c	12 (24.0)
T2a	21 (42.0)
T2b	7 (14.0)
T2c	10 (20.0)
Biopsy grade group, *n* (%)	
Grade group 1	19 (38.0)
Grade group 2	16 (32.0)
Grade group 3	11 (22.0)
Grade group 4	4 (8.0)
NCCN risk group, *n* (%)	
Low risk	14 (28.0)
Favorable intermediate risk	13 (26.0)
Unfavorable intermediate risk	16 (32.0)
High risk	7 (14.0)
ASA score, *n* (%)	
≤2	44 (88.0)
≥3	6 (12.0)
CCI, *n* (%)	
0~1	47 (94.0)
≥2	3 (6.0)
Surgical history, *n* (%)	
Appendectomy	2 (4.0)
Cholecystectomy	4 (8.0)
Colectomy	3 (6.0)
Gastrectomy	2 (4.0)
Hepatectomy	2 (4.0)
Ileus	1 (2.0)
Inguinal/umbilical hernia repair	6 (12.0)
Nephrectomy	1 (2.0)
Transurethral resection of the prostate	1 (2.0)

All surgeries were completed without conversion. The median (IQR) operation time was 205.0 (82.5) min, the median (IQR) docking time was 30.0 (15.0) min, and the median (IQR) console time was 120.0 (80.5) min. The median (IQR) estimated blood loss (EBL) was 50.0 (137.5) mL. The median hospital stay was 8.0 (6.0) days, and 13 (26.0%) patients accepted the neurovascular bundle (NVB) sparing (Table 2).

**Table 2:** Perioperative and postoperative data of patients (*N*=50)

Variables	Results
Operation time, min	205.0 (82.5)
Docking time, min	30.0 (15.0)
Console time, min	120.0 (80.5)
Estimated blood loss, mL	50.0 (137.5)
Transfusion, *n* (%)	0 (0)
Neurovascular bundle sparing, *n* (%)	13 (26.0)
Pain medication after discharge, *n* (%)	
Opioids	37 (74.0)
NSAIDS only	13 (26.0)
Catheter indwelling time, days	14.0 (5.3)
Hospital stays, days	8.0 (6.0)

Positive surgical margins (PSM) were detected in 5 patients (10.0%), and 19 patients (38.0%) experienced pathologic upstaging with grade group compared to the preoperative data (Table 3).

**Table 3:** Pathological data of patients (*N*=50)

Variables	Results
PSM, *n* (%)	5 (10.0)
PSM risk^a^, *n* (%)	
Low risk	9 (18.0)
Favorable intermediate risk	8 (16.0)
Unfavorable intermediate risk	18 (36.0)
High risk	15 (30.0)
Biopsy grade group, n (%)	
Grade group 1	11 (22.0)
Grade group 2	20 (40.0)
Grade group 3	13 (26.0)
Grade group 4	3 (6.0)
Grade group 5	3 (6.0)
Upgrade	19 (38.0)
Pathological stage, *n* (%)	
T2a	17 (34.0)
T2b	3 (6.0)
T2c	23 (46.0)
T3a	2 (4.0)
T3b	5 (10.0)

^a^Low risk: grade group 1 and PSA<10; favorable intermediate risk: grade group 2 and PSA<10; unfavorable intermediate risk: grade group 3 or PSA≥10; and high risk: PSA > 20 ng/ml or T stage >2 or grade group≥4

The median (IQR) follow-up was 10.5 (6.0) months (Table 4). The respective continence rates were 40.9%, 63.6%, 88.4%, and 97.7% at 1, 3, 6, and 12 months after surgery. Only 1 (2.0%) patient experienced biochemical recurrence 9 months after surgery.

**Table 4:** Functional and oncological outcomes of patients (*N*=50)

Variables	Results
Follow-up, months	10.5 (6.0)
Continence rate, %	
1 months	40.9
3 months	63.6
6 months	88.4
12 months	97.7
Biochemical recurrence, *n* (%)	1 (2.0)

The overall complication rate was 24% (Table 5). One urinary tract injury case and one rectal injury case were immediately identified and managed intraoperatively. A Grade IIIa complication in one patient who required urethral dilation for a vesicourethral stricture. Two Grade II cases of poor wound healing were treated with infrared light irradiation-assisted rehabilitation. Antibiotics were used to treat patients with urinary tract or wound infections. One patient experienced a Grade IV complication secondary to acute respiratory distress syndrome (ARDS) requiring ICU care for respiratory support. One Grade II complication was for readmission to manage a prolonged wound infection.

**Table 5:** Complications and readmission (*N*=50)

Variables	Results
Total complications cases, *n* (%)	12 (24.0)
Complication type, *n* (%)	
Acute respiratory distress syndrome	2 (4.0)
Poor wound healing	2 (4.0)
Rectal injury	1 (2.0)
Urethral injury	1 (2.0)
Urinary tract infection	2 (4.0)
Vesicourethral stenosis	1 (2.0)
Wound infection	3 (6.0)
Clavien-Dindo grade, *n* (%)	
I	2 (4.0)
II	7 (14.0)
IIIa	1 (2.0)
Iva	2 (4.0)
Readmission, *n* (%)	1 (2.0)

**Fig. 3:**
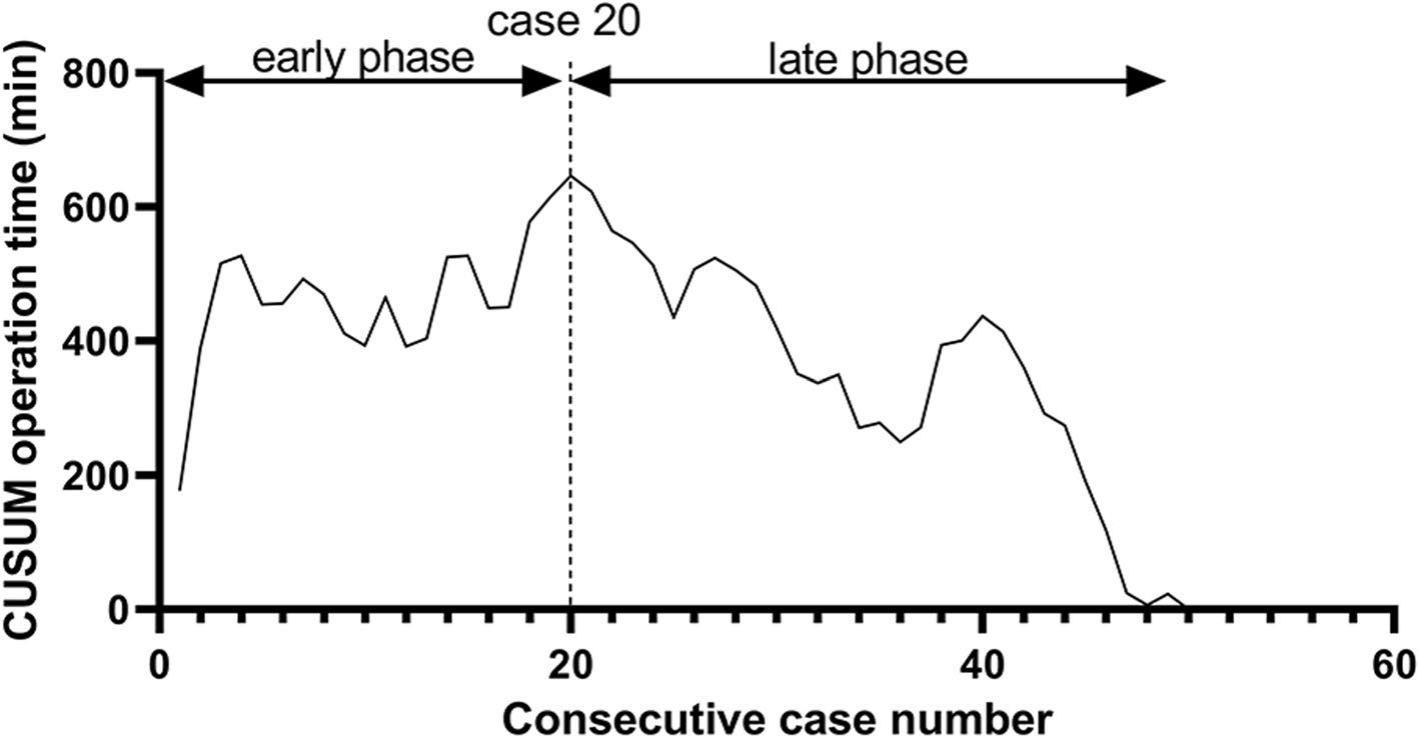
The CUSUM analysis of the operation time (OT)

The peak of the CUSUM curve was considered the breaking point for surgeons to master a new technique, and 20 cases was regarded as the inflection point in this study (Fig. 3). The No.1~No.20 cases were defined as “early phase,” while the No.21~No.50 cases were defined as “late phase.” “Late phase” cases required less time for each step of the procedure and experienced less EBL [175.0 (225.5) mL vs 50.0 (50.0) mL, *p*=0.023] (Table 6). The patient demographics, pathological outcomes, and complications did not reveal significant differences between early and late phase cases (Additional files 1, 2, 3).

**Table 6:** The comparison between “early phase” and “late phase”

Variables	Early phase (*N*=20)	Late phase (*N*=30)	*p* value^*^
Operation time, min	235.0 (131.3)	200.0 (72.5)	**0.015**
Docking time, min	40.0 (20.0)	28.5 (13.5)	**0.005**
Console time, min	165.0 (80.0)	100.0 (55.0)	**0.001**
Estimated blood loss, mL	175.0 (225.5)	50.0 (50.0)	**0.023**
Catheter indwelling time, days	18.0 (8.0)	14.0 (1.3)	**0.013**
Hospital stays, days	7.0 (3.8)	8.5 (6.5)	0.157
Continence rate, %			
1 months	31.6	48.0	0.948
3 months	42.1	80.0	**0.010**
6 months	78.9	95.8	0.086
12 months	94.7	100.0	0.442
Biochemical recurrence, *n* (%)	1 (5.0)	0 (0)	0.311

^*^p values<0.05 were considered statistically significant

## Discussion

This current study is one of the largest retrospective studies to focus on sp-pRARP using the da Vinci XI system, as well as the first to focus on the sp-pRARP learning curve. Open perineal RP was abandoned and replaced by the retropubic approach because of the limited perineal anatomy, high incidence of complications, and the advent of minimally invasive laparoscopic surgeries. Robotic surgical platforms have brought an improved three-dimensional view of the surgical field and flexible robotic instrumentation allowing surgeons to accomplish complex maneuvers in smaller spaces thus creating opportunities to develop novel methods such as ours.

Abdominal/pelvic surgical history, such as inguinal hernia repair (IHR), have proposed significant challenges for robot-assisted RP due to the adhesions and distortion of anatomic planes [19], and obscuring the pelvic lymph node dissection (PLND) [20]. The adhesions in the inguinal area induced by IHR would make it difficult to place trocars in the appropriate position during retropubic RP, lengthening the trocars placement time and the prostatectomy time [21]. The perineal approach offers another surgical option when faced with a patient with extensive prior abdomino-pelvic surgical history, eliminating the concern of adhesiolysis. In our study, there was no statistically significant difference in terms of the parameters between patients with and without a history of IHR (Additional file 4). The results might be explained by the small number of patients with IHR history, but we could still presume that sp-pRARP might be an alternative for individuals who were not candidates for retropubic RARP.

In terms of perioperative outcomes such as operative time and EBL, the results were comparable to other researches [8–11]. However, the length of hospital stays [8.0 (6.0) days] was longer than previous reports, which were mostly less than 2 days [8, 10, 11]. The use of new technology made us choose more conservative discharge criteria so that patients could have their pelvic drains removed and get a better recovery during their hospital stays. Besides, the observed complications such as wound infection, poor wound healing, urinary tract infection, and the rare ARDS, which required extended antibiotic treatment or respiratory support, also lengthened the hospital stays. With increased surgical proficiency and experience in postoperative management, we believe that the hospital stay could be reduced in future practices.

Bilateral pelvic lymph node dissection (PLND) is a critical procedure that should not be overlooked and is challenging to perform in the sp-pRARP. The staging information gained from PLND comes at the risk of prolonged surgical times and lymphorrhagia. According to the American Association of Urology and European Association of Urology [22, 23], PLND should be performed when the predicted probability of lymph node metastasis is greater than 2% or greater than 5%, and patients with low risk do not require PLND. Ramirez et al. [24] firstly described the technique of PLND in sp-pRARP on the da Vinci SP platform. Tugcu et al. [25] also introduced the Tugcu-Bakirkoy technique to complete the same procedure on the da Vinci XI system. The majority of patients in our series had low to intermediate risk cancers, and preoperative PET/CT examination was used for patients who had a relatively high risk of lymph node metastasis. Patients with lymph node metastases determined by MRI or PET/CT were excluded. Due to the unfamiliarity and inherent difficulties of PLND in sp-pRARP, and the low possibility of lymph node metastases shown by preoperative radiological imaging, PLND was not performed in this investigation.

PSMs have been identified as an independent predictor of biochemical recurrence (BCR) and may lead to subsequent adjuvant or salvage therapies (radiotherapy with or without androgen deprivation therapy). According to a multicenter study, the overall PSM rate for RARP was 13.6%, while the PSM rates for the pT2 and pT3 stages were 9.45% and 37.2%, respectively [26]. The PSM rate of p-RARP ranged from 8.4 to 65.4% in known case series [8–11]. In this study, 5 (10%) patients experienced a PSM. Only one patient had a BCR during our short follow-up period and required salvage radiotherapy. Additional longitudinal follow-up analyses may reveal more complete oncologic outcomes in the future.

Continence status is an important functional outcome parameter after RARP. Several attempts have been introduced in order to provide better functional outcomes for patients, including bladder neck preservation, neurovascular bundle (NVB) sparing, posterior reconstruction, and/or anterior suspension and bladder neck plication [5]. The “Retzius sparing” and “PERUSIA” techniques were also applied in recent years for earlier continence recovery [27–30]. Meta-analysis has supported that RARP is associated with excellent 12-month continence, with urine continence recovery rates ranging between 84 and 97% [31]. There are 26% patients underwent NVB sparing intraoperatively, and the 12 months following indicated a 97.7% recovery rate for postoperative continence. We feel the post sp-pRARP continence recovery may be improved because the perineal approach allowed for less injury to the auxiliary pudendal arteries and dorsal veins, as well as improved preservation of the endopelvic fascia.

As anticipated when adopting a new technique, the incidence of complications was higher than expected but fortunately not severe. The risk of carbon dioxide (CO_2_) embolism, although has a low incidence, has an extremely high fatality rate in laparoscopy [32]. Bao et al. [33] reported one case of CO_2_ embolism during laparoscopic RP. But in the reported RARP case series, this severe complication has not been reported. In our study, two patients experienced ARDS due to CO_2_ embolization, which was most likely caused by the intelligent pneumoperitoneum Airseal® insufflator. The insufflator maintained a steady pressure by a continuous infusion of CO_2_, potentially preventing air leaks during specimen collection or instrument/lens changes that could disrupt the flow of the process. During a prolonged procedure, the charged CO_2_ could be absorbed in considerable quantities by the surrounding tissue or ruptured venous plexus, resulting in CO_2_ embolism and manifesting as decreased percutaneous oxygen saturation (SpO_2_) and end-tidal carbon dioxide (ETCO_2_), hypoxemia, hypercapnia, increased airway resistance, and hemodynamic abnormalities. Intraoperative precautions are also necessary. When a venous plexus rupture is found, the surgery should be halted and the CO_2_ insufflation pressure must be reduced. And the patient’s SpO_2_ changes should be monitored closely throughout the surgical process.

In addition, poor wound healing and wound infection were observed in our study. It was considered that mini-invasive techniques could decrease the rate of wound-related complications and unplanned hospital visits because of less blood loss and less ischemic suture resulting in lower systemic stress, higher immune level and less local tissue injury [34, 35]. Since incision length is one of the factors associated with incision infection [35], the purpose-built SP system allows for a shorter incision, which may provide a better protection of the local tissue and has the potential benefit for reducing complications, and the reported research showed the rate of wound incision was indeed at a low level (less than 5%) [7, 8, 11]. But in our study, the incidence of wound-related complications was higher. The long operation time and the pressing of the port to the skin around the incision may cause local ischemia. And the perineal incision is located in a moist area near the anus, making it susceptible to fecal contamination and increase the risk of wound infection or poor healing. Inadequate drainage can also delay the healing of an incision. Therefore, emphasis should be placed on the intraoperative incision protection, postoperative sterilization of the incision site, and the preservation of drainage to facilitate wound healing.

Our research is the first to examine the learning curve of da Vinci XI sp-pRARP by using the CUSUM method. The CUSUM plot demonstrated that 20 cases was the critical cutoff point for the learning curve of an experienced robotic/laparoscopic surgeon. Moreover, comparisons between the two phases revealed that proficiency had improved significantly in the “late phase”. The operating time, docking time, and console time were all noticeably shorter in the “late phase” compared to the “early phase”, and the EBL was also noticeably reduced. Although the operation time for sp-pRARP was still significantly longer compared to mp-RARP, our learning curve showed a significant improvement with the accumulation of experience. Due to a greater understanding of the anatomy of the perineal region, surgeons may be able to operate more skillfully and with less damage to the surrounding blood vessels after a certain amount of experience and practice. Regarding continence, the difference was only observed during the initial three months, and it decreased as time passed.

A thorough understanding of the anatomy of the perineal area is one of the most important factors for the success surgery. Preoperative suprapubic ultrasonography is required to evaluate the prostate size and pelvic anatomy. The probe-divergence angle (PDA), which means the angle between the vertical axis and the oblique plane of the probe while it was being directed at the base of prostate, was necessary to describe the deepness of prostate. The cases with smaller size of prostate and lower location (nearer to the perineum in ultrasonography) were considered to be “easier” [36]. Whereas an increase in BMI has little effect on the efficacy of the procedure, as less fat accumulates in the perineal area [37].

Compared to SI system, the laser location and the capacity to automatically calculate the ideal surgical posture of the robotic arm of the XI system enable more precise automatic docking and reduce docking time. It also equipped thinner and more flexible robotic arm joints allowing for a broader range of motion, ensuring a safe distance between the arm and the patient and preventing friction between the arm and the abdominal wall of the patient. The greater resolution lens provides a clearer view of the anatomy and permits better differentiation of anatomical levels, particularly in the posterior plane of the prostate, in order to prevent rectal injuries [38]. Therefore, the sp-RARP on XI systems delivers a significant improvement over the SI system in terms of operation time and EBL [6, 12]. But there is still a gap with the purpose-built SP system, which incorporated an extra joint to create an “elbow” so that the instruments could triangulate around the target anatomy and reduce the incidence of instruments clashing [7]. The SP system also allowed a single arm to have 360° of anatomical access to make the operation more accurate. And it was considered allowing for a smaller incision and potentially reducing the incidence of postoperative complications, including infectious incisions.

Our study has several limitations. First, the conclusions were derived from a small-sample retrospective study, and potential selection bias may have affected the reliability of the final results. Second, the short follow-up period prevented the conclusion of long-term postoperative functional recovery and oncological outcomes. Although additional research is needed to verify the long-term safety and efficacy of sp-pRARP, it may offer another surgical option to patients with complicated abdominal/pelvic histories.

## Conclusions

Sp-pRARP using the da Vinci XI system proved to be a safe and feasible alternative surgical method for treating prostate cancer. It can be an alternative for patients with a history of complex abdominal/pelvic surgery, but some special complications require our attention. According to the CUSUM plot, 20 cases is considered the learning curve threshold before one becomes more proficient and experiences less blood loss. Additional prospective multi-center clinical trials including more patients should be completed to validate sp-pRARP outcomes.

## Supplementary Information

**Additional file 1. **Demographic of patients in “early phase” and “late phase”.

**Additional file 2. **Postoperative and pathological data of patients in “early phase” and “late phase”.

**Additional file 3. **Complications of patients in “early phase” and “late phase”.

**Additional file 4. **Perioperative outcomes between patients with and without IHR history.

## Data Availability

The datasets used and/or analyzed during the current study are available from the corresponding author on reasonable request.

## Abbreviations

ARDS: Acute respiratory distress syndrome
ASA: American Society of Anesthesiologists
BCR: Biochemical recurrence
BMI: Body mass index
CCI: Charlson comorbidity index
CO_2_: Carbon dioxide
CUSUM: Cumulative sum
EBL: Estimated blood loss
ETCO_2_: End-tidal carbon dioxide
IHR: Inguinal hernia repair
Mp-RARP: Multiport robot-assisted radical prostatectomy
NVB: Neurovascular bundle
PCa: Prostate cancer
PDA: Probe-divergence angle
PLND: Pelvic lymph node dissection
PSM: Positive surgical margin
RP: Radical prostatectomy
Sp-eRARP: Single-port robot-assisted extraperitoneal radical prostatectomy
SpO_2_: Percutaneous oxygen saturation
Sp-pRARP: Single-port robot-assisted perineal radical prostatectomy
